# Solubilization of Reactive Red 2 in the Mixed Micelles of Cetylpyridinium Chloride and TX-114

**DOI:** 10.3390/molecules28093952

**Published:** 2023-05-08

**Authors:** Tayyba Yaqoob, Saadia Shaukat, Rasha Alonaizan, Ramzan Ullah, Imran Khan, Muhammad Faizan Nazar, Hafiz Muhammad Abd Ur Rahman

**Affiliations:** 1 Department of Chemistry, Government College Women University Sialkot, Sialkot 51310, Pakistan; 2 Zoology Department, College of Science, King Saud University, Riyadh 11451, Saudi Arabia; 3 Department of Physics, Rawalpindi Women University, Rawalpindi 46300, Pakistan; 4 Acoustophoreticn Single Embryo Characterization Laboratory (ASECL), Department of Biomedical Engineering, School of Medicine, Keimyung University, 1095 Dalgubeol-daero, Dalseo-gu, Daegu 42601, Republic of Korea; 5 Department of Chemistry, University of Education Lahore, Multan Campus, Lahore 60700, Pakistan

**Keywords:** reactive red 2, ionic surfactant, nonionic surfactant, mixed micelles, binding coefficient, conductometry, spectroscopy

## Abstract

Owing to their surface active properties, surfactants have numerous applications in different fields of life. In the present research work, the solubilization of reactive red 2 (RR2) has been studied in single and mixed micellar systems (MMS) using UV-visible spectroscopy and electrical conductivity measurements. The interaction of RR2 with ionic micelles of cetylpyridinium chloride (CPC) was investigated. In order to probe the interaction of RR2 in MMS, mixtures of CPC and TX-114 (Triton X-114, a nonionic surfactant) were used. UV-visible spectroscopy has been used to obtain the degree of solubilization of RR2 in terms of the partition coefficient (*K_c_*) and Gibbs free energy of partitioning (Δ*G****^°^****_p_*). Electrical conductivity data have been employed to detect the critical micelle concentration (CMC) of the surfactant systems in the presence of RR2 and, accordingly, to calculate the thermodynamic parameters of the micellization. From the obtained data, it is concluded that the micellization is spontaneous at all studied temperatures. Moreover, the micellization was observed to be driven by both enthalpy and entropy. The results also indicated that MMS have better solubilizing power than single micellar solutions.

## 1. Introduction

Surfactants are organic compounds with a characteristic molecular structure consisting of a hydrophilic group that has a strong affinity for polar media and a hydrophobic group that has a strong attraction for non-polar media. Such substances have the ability to alter the structure of the polar solvent via their hydrophobic groups due to their amphipathic nature, and they enhance the Gibbs energy of that particular system. During this process, the system rearranges itself in such a way as to reduce the contact among the hydrophobic species and polar solvent [1]. Surfactants, or surface-active agents, change the surface properties and reduce the surface tension of a system when present at low concentration in a system. They start forming self-aggregates called micelles at a specific concentration called the critical micelle concentration (CMC) [2]. Due to these properties, surfactants are extensively used in emulsifying, washing, wetting, and dispersing. Many surfactants, such as alkyl benzene sulfonates and sulfates are used in laundry detergents, and in household and personal care products [3].

Depending upon the types of head groups, surfactants are divided into two major groups, i.e., ionic surfactants and nonionic surfactants. A surfactant comprised of a charged hydrophilic head is termed an ionic surfactant. Ionic surfactants are further classified as anionic, cationic, and zwitterionic [4]. Ionic surfactants are mostly used in powder and liquid form as laundry detergents, soap formation, and for the disruption of various proteins. The hydrophilic head group in nonionic surfactants bears no apparent ionic charge [4]. Nonionic surfactants have their applications mostly in the food, cosmetic and pharmaceutical industries due to their less toxic nature. Gemini surfactants belong to another emerging class of surfactants [5,6,7]. These surfactants have attained the attention of researchers due to their numerous applications [5,6,7,8] in different fields. These surfactants have a very low CMC and a greater solubilization power as compared to the conventional surfactants [9,10].

The ability of a surfactant to form micelles is crucial for its applications in detergency and the solubilization of non-polar substances. Micelle formation occurs due to non-covalent interactions of the non-polar portion of the surfactant molecules, i.e., via Van der Waals forces and hydrophobic–hydrophobic interactions. Surfactants are known to form micelles of different shapes, such as spherical, cylindrical and lamellar. A surfactant micelle is identified by three regions, i.e., outer region, core and the palisade region [11]. Due to their hydrophilic outside and hydrophobic inside, micelles are regarded as equivalent to a biological membrane. They are capable of interacting with both polar and non-polar molecules. The binding constant, partition coefficient, and changes in the different thermodynamic parameters of micellization can be used to measure the strength of such interactions [12,13].

Generally, nonionic surfactants have better solubilizing power than ionic surfactants. However, temperature has a significant impact on the phase behavior of nonionic surfactants containing oxyethylene groups. An increase in temperature causes phase separation in a nonionic surfactant solution at a temperature called the cloud point. At the cloud point, a single micellar solution is separated into two different phases. One phase contains a greater concentration of the surfactant and the other one possesses its low concentration [14]. In order to avoid phase separation, nonionic surfactants can be mixed with ionic surfactants to form mixed micelles. Mixed micelles have the ability to enhance the solubilization of insoluble or partially soluble substances. In this regard, mixed micelles are more beneficial than the micelles formed by a single surfactant. Due to the stable electrical structure of a mixed micelle, they show more exclusive and superior qualities than single micelles. The mixing of nonionic surfactants with ionic surfactants may cause changes in the shape of the micelle; an increase in their solubilization tendency; decreased repulsion between the ionic head groups; and enhancement of the hydrophobicity of the core of the micelles [15,16].

Dyes belong to the unsaturated class of organic compounds that absorb light in the visible region to impart the color. The chromophoric part of a dye couples with resonating electrons and auxochrome is the substituent, which is attached to the chromophore and increases the absorption intensity of the chromophore [17]. Dyes are used in many industries, such as paper, pulp, plastic, paints, etc. Several issues arise from the increased use of dyes in everyday life. The discharge of dyes into rivers and lakes causes serious damage to fauna and flora. The release of dye into the hydrosphere causes an undesirable color and smell in drinking water [18]. Water contaminated with dye causes many problems to human life. Dyes can badly damage humans as they have a negative impact on the human brain, may cause liver dysfunction, and affect the central nervous system, kidneys and reproductive system. Hence it is extremely important to remove dyes from effluent water. Micellar solubilization is one of the important methods used for the removal of effluent dyes from waste water, especially weakly soluble dyes [14,15,17,18,19,20,21,22]. In this regard, the study of dye–surfactant interactions is very important.

**Figure 1:**
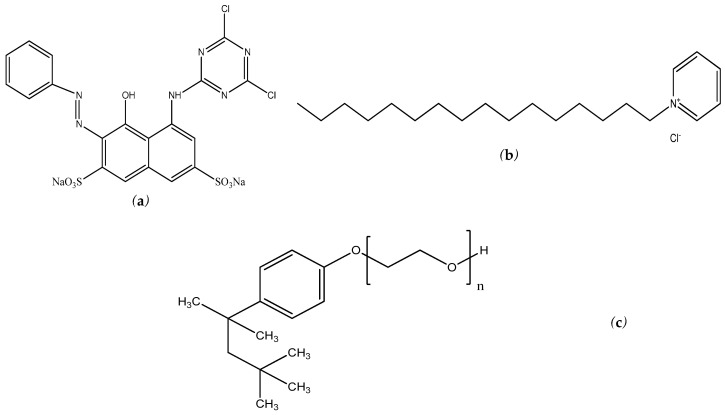
Chemical structure of (**a**) RR2; (**b**) CPC; and (**c**) TX-114.

The present work reports on the interaction of reactive red 2 (RR2), an azo dye that is used in dying textiles, with mixed micelles of TritonX-114 (TX-114, a nonionic surfactant) and cetylpyridinium chloride (CPC, a cationic surfactant) in aqueous solution. Different thermodynamic parameters, such as the change in the free energy of micellization ($\Delta G_{mic}^{{}^{\circ}}$), the change in the enthalpy of micellization (Δ*H_m_*), and the entropy of micellization (Δ*S_mic_*) for mixed surfactant systems have been obtained in the presence of RR2 dye. The partition coefficient ($K_{x}$) and binding constant ($K_{b})$ were determined using UV-visible spectroscopy. Figure 1 shows the molecular structure of RR2, CPC, and TX-114.

## 2. Results and Discussion

### 2.1. UV-Visible Spectroscopy

The binding constant, *K_b_*, was calculated from the Benesi–Hildebrand equation (Equation (1)) [23]. A plot between *dC_d_*/Δ*A* and 1/$C_{s}^{mo}$ gave the value *K_b_*.

(1) $$\frac{dC_{d}}{\Delta A}=\frac{1}{K_{b}\Delta \varepsilon C_{s}^{mo}}+\frac{1}{\Delta \varepsilon }$$

where *C_d_* is the concentration of dye, RR2 and Δ*A* is the difference of the absorbance between the RR2–CPC surfactant complex and RR2. Δ*ε* is the difference in the absorption coefficient of the complex and dye (Δ*ε* = *ε* − *ε˳*).

The partition coefficient, *K_c_*, a quantitative measure of the degree of solubilization, was quantified using the Kawamura equation (Equation (1)) [24]:

(2) $$\frac{1}{\Delta A}=\frac{1}{K_{c}\Delta A_{\infty }\left(C_{d}+C_{s}^{mo}\right)}+\frac{1}{\Delta A_{\infty }}$$

In Equation (1), *C_d_* is the concentration of RR2 in mol/dm^3^, *C_s_^mo^* is the micellar concentration of the surfactant, Δ*A* and Δ*A*_∞_ represent the values of the differential absorbance at the experimental and infinite concentration of the surfactant. *K_c_* is the partition constant in units of dm^3^/mol.

The value of *K_c_* is used for the calculation of the dimensionless partition coefficient (*K_x_*) [25] using Equation (3):

(3) $$K_{x}=K_{c}n_{W}$$

Here, *n_w_* represents the number of moles of water per dm^3^, i.e., 55.5 moles/dm^3^.

From the values of *K_b_* and *K_x_*, the Gibbs free energy of interaction (Δ*G_b_*) and the Gibbs free energy of the transfer of dye from the bulk aqueous phase to the micellar phase (Δ*G_p_*) were determined using the relation [26]:

(4) ΔG=−RTlnK

where *R* is the general gas constant and *T* is the absolute temperature.

**Figure 2:**
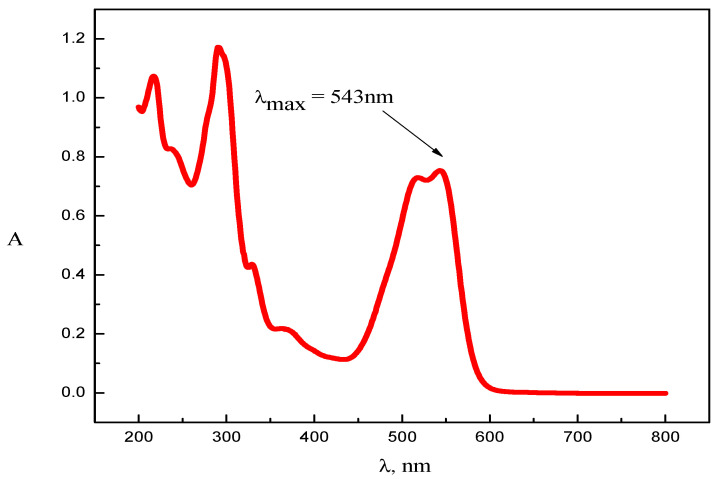
Absorption spectrum of RR2 dye in distilled water.

Figure 2 presents the simple absorbance, A, spectrum of pure RR2 dye in water. The optimum dye concentration of 1.00 × 10^−4^ M was kept constant in all experiments. As evident from Figure 2, the spectrum of the RR2 dye shows peaks in both the UV and visible regions. Chromophoric azo bonds, which impart color to the dye, are responsible for the transitions in the visible region, whereas both the aryl- and naphthalene-like moieties are responsible for the peaks in the UV region [27].

**Figure 3:**
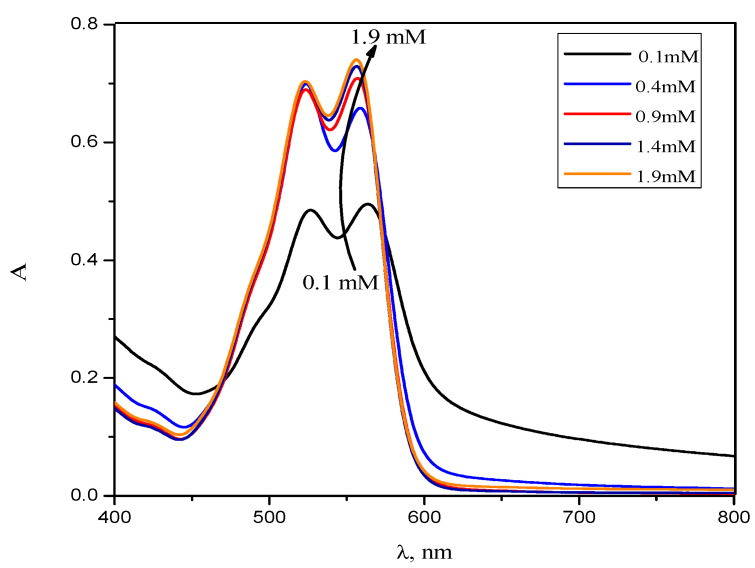
Simple absorbance spectra of RR2 in the presence of different concentrations of CPC in an aqueous solution.

Figure 3 and Figure S1 (Supplementary Materials)  display the absorption spectra of the RR2 dye in the presence of different concentrations of CPC in an aqueous solution. RR2 showed a maximum absorption at 543 nm in an aqueous solution. However, in the presence of CPC, it showed a maximum absorption at 564 nm. This indicates the presence of electrostatic forces between the oppositely charged dye and surfactant head groups [19,28,29]. The interaction takes place between the sulfate group of the RR2 and the cationic head group of the surfactant, which reduces the repulsion between the CPC head groups and increases the solubilization of the dye into the micelles. Due to electrostatic forces, the energy gap between the n and $\pi^{*}$ orbitals was increased and, therefore, the wavelength of the maximum absorption, λ_max_, was shifted to a higher value [12,28]. This bathochromic shift can also be ascribed to the change in the micro environment of the dye from polar (water) to less polar or non-polar (micelles) upon solubilization [12,28,29].

**Figure 4:**
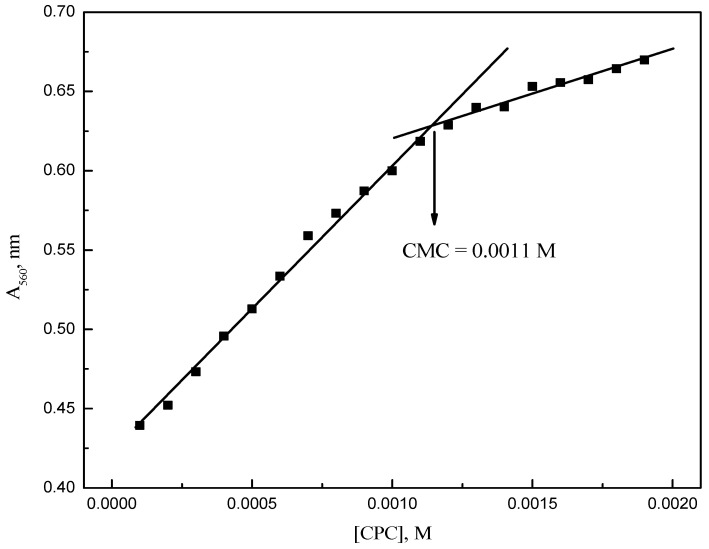
Variation of the RR2 absorbance at 560 nm with the CPC concentration.

The plot of the absorbance at λ_max_, A_560_, as a function of the molar concentration of CPC is shown in Figure 4. A CMC value of 0.0011 M of CPC in the presence of RR2 was obtained from the spectroscopic data.

**Figure 5:**
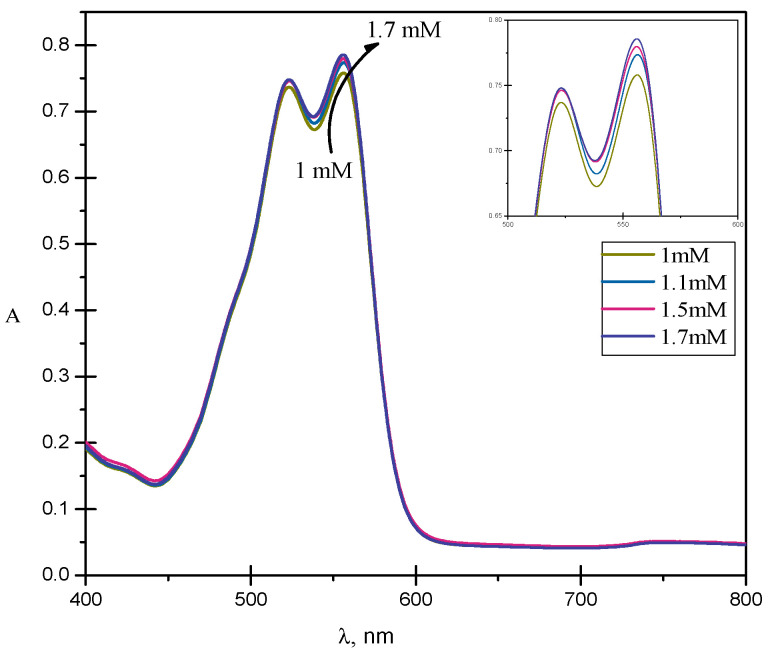
Differential absorbance spectra of RR2 as a function of the different concentration of CPC.

Figure 5 and Figure S2 (Supplementary Materials) represent the differential absorbance spectra of the RR2 as a function of post-micellar concentrations of CPC. The data of the differential absorbance were used to calculate the binding constant, K_b_; partition constant, Kc; the Gibbs free energy of binding, Δ*G_b_*; and the Gibbs free energy of partitioning, Δ*G_p_*, of the RR2 in the CPC micelles.

**Figure 6:**
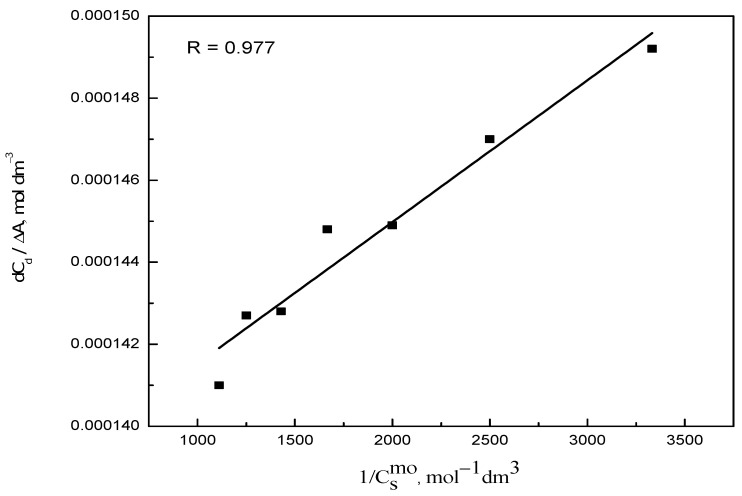
Plot for the calculation of the binding constant, *K_b_*, of the RR2–CPC system.

The Benesi–Hildebrand equation (Equation (1)) was used to calculate the binding constant of RR2 with the micelles of CPC. The linearity of the data was tested by drawing the plot between *dC_d_*/Δ*A* and *1/*$C_{s}^{mo}$ (Figure 6). The value of the binding constant was determined from the value of the slope and intercept.

**Figure 7:**
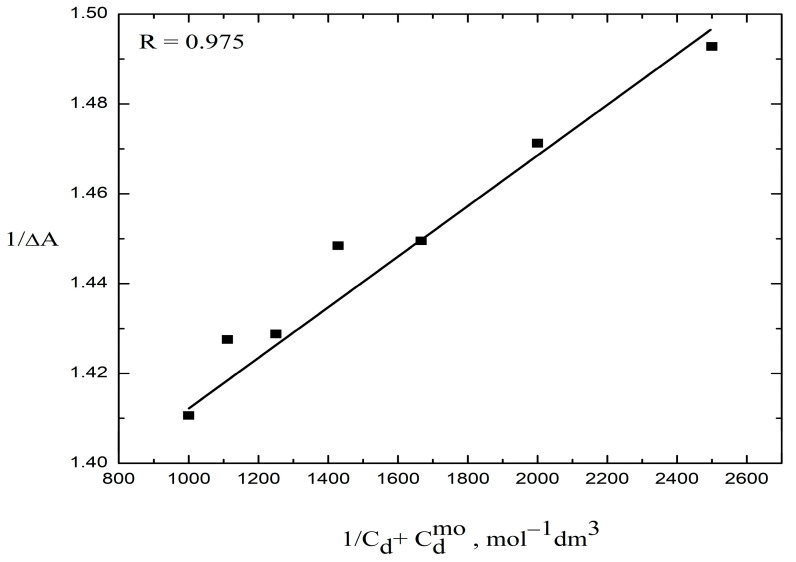
Plot for the calculation of the partition coefficient, *Kc*, for the RR2–CPC system.

The partitioning of the dye in the aqueous solution and in the hydrophobic core of the micelles was determined using the partition law, according to the Kawamura model (Equation (2)). Partitioning of the dye is determined in the post micellar region from the differential absorption spectra of different solutions of surfactants in the presence of RR2. A graph is plotted between 1/Δ*A* and 1/($C_{d}+C_{s}^{mo}$) (Figure 7) from which the value of the slope and intercept is used to calculate the partition coefficient. The Gibbs free energy of partitioning (Δ*G_p_*) is determined from the value of *K_x_.* All the determined parameters of the interaction of RR2 with the ionic micelles of CPC are given in Table 1.

Similarly, differential absorbance spectroscopy was employed to estimate the *K_b_* (Figure S3 of Supplementary Materials) and *Kc* (Figure S4 of Supplementary Materials) of the RR2 in TX-114 micelles in an aqueous solution. The values of *K_b_* and *K_c_* were further used to calculate the Δ*G_b_*, and Δ*G_p_* of the RR2 in aqueous solution TX-114 micelles. The obtained values of *K_b_*_,_ *Kc*, Δ*G_b_*, and Δ*G_p_* are presented in Table 1.

From the magnitudes of *K_b_* and *K_x_*, it is reasonable to infer that RR2 dye can be solubilized efficiently into CPC and TX-114 micelles. The relatively large value of *K_b_* for the RR2–CPC as compared to the RR2–TX-114 indicates that RR2 binds strongly with CPC micelles. Similarly, the higher value of the *K_x_* of RR2–CPC indicates that CPC micelles solubilize RR2 more efficiently than TX-114. It can further be inferred from the *K_x_* results for both systems (RR2–CPC and RR2–TX-114) that RR2 prefers to move from an aqueous medium to a more hydrophobic micellar phase. In addition, the high value of *K_x_* suggests that the RR2 molecules reside in the palisade layer [17]. The negative values of Δ*G_b_* and Δ*G_p_* indicate that both the binding and solubilization of RR2 in the CPC and TX-114 micelles occur spontaneously.

**Figure 8:**
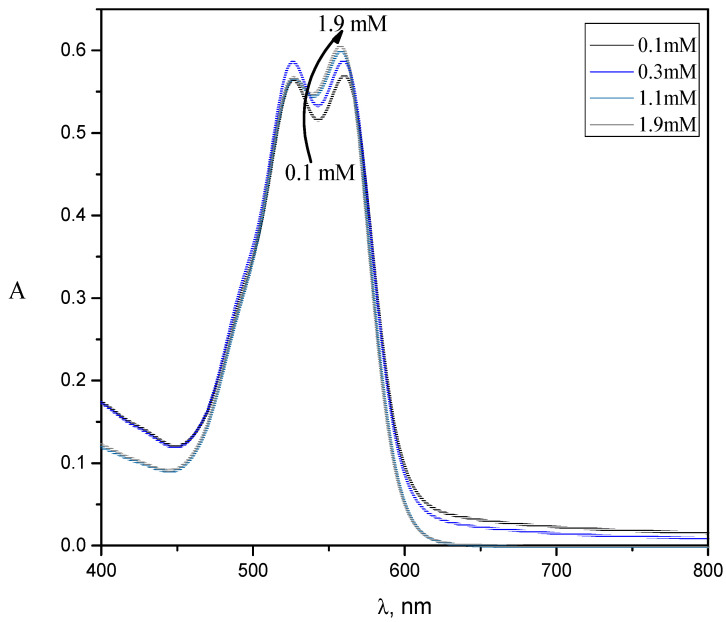
Simple absorbance spectra of RR2 in the presence of 0.13 mM TX-114 in the CPC/TX-114 mixed micelles at different concentrations of CPC.

Figure 8 and Figure S5 (Supplementary Materials) show the spectra of RR2 in the presence of 0.13 mM TX-114 in the CPC–TX-114 mixed micellar system (MMS). It is evident from the spectra that the absorbance increases by increasing the concentration of CPC. The maximum absorbance was observed at 560 nm.

**Figure 9:**
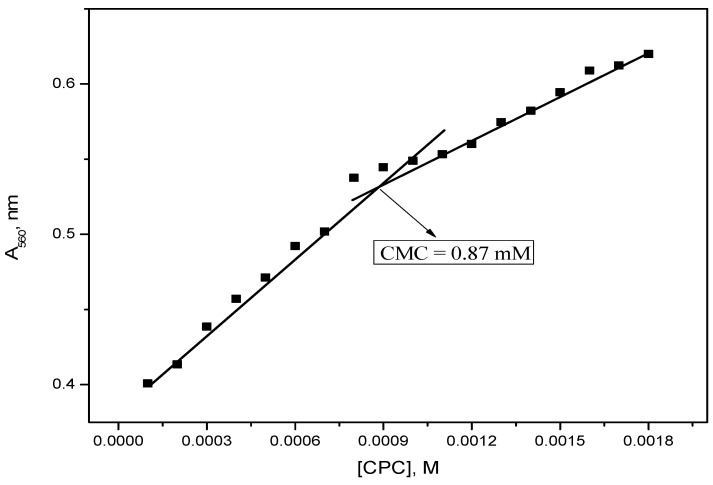
Variation of the RR2 absorbance at 560 nm with the CPC concentration in the presence of 0.13 mM TX-114.

Figure 9 shows the plot of absorbance of RR2 in the presence of 0.13 mM TX-114 in the MMS. The CMC value of 0.87 mM of the MMS in the presence of RR2 has been determined from the break point of the absorbance of RR2 versus the molar concentration of CPC in the presence of a constant concentration of TX-114. Generally, nonionic surfactants decrease the repulsive forces between the ionic head groups of surfactants. For this reason, the CMC decreases and the solubilization power of a surfactant increases [30]. To study the solubilization power of mixed micelles, different concentrations of TX-114 were used.

**Figure 10:**
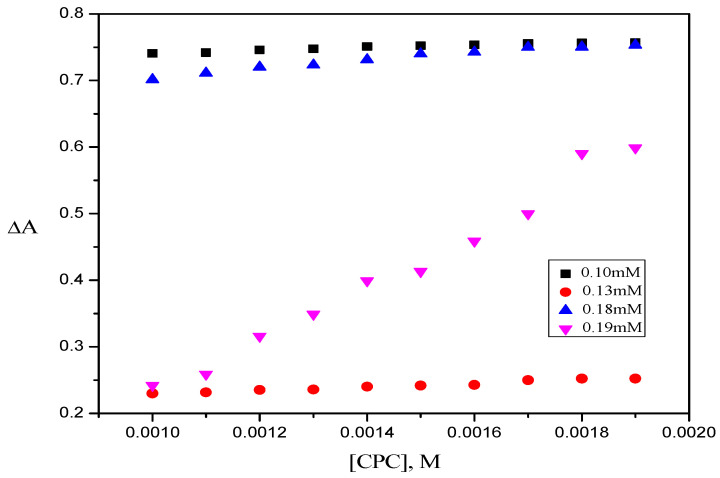
Plot of the differential absorbance of RR2 dye in the presence of different concentrations of CPC for four different concentrations of TX-114.

The binding and partition coefficient values of the MMS have been calculated from the differential absorbance spectroscopy. As mixed micellar systems are expected to have more solubilizing power, the solubilization of the RR2 dye is checked at different concentrations of TX-114. The differential absorbance of RR2 dye, ΔA, as a function of different concentrations of CPC for four different concentrations of TX-114 i.e., 0.10 mM, 0.13 mM, 0.18 mM, and 0.19 mM, is shown in Figure 10.

**Figure 11:**
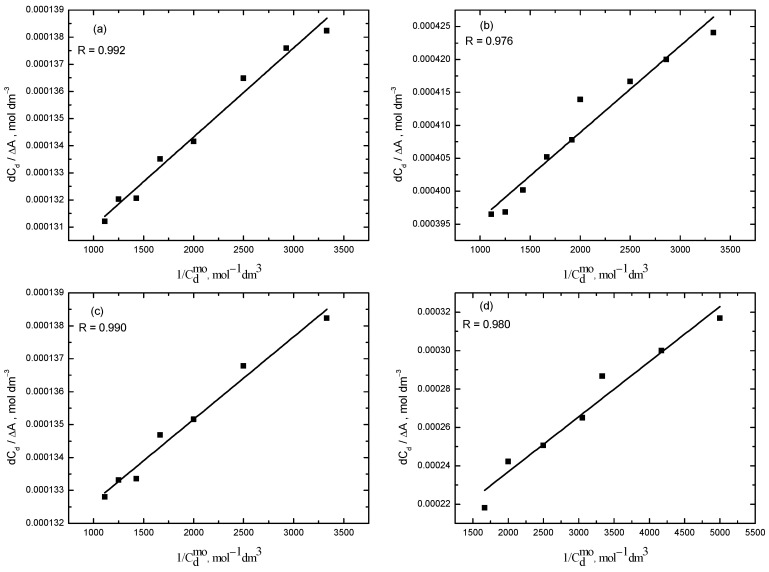
Plots for calculation of the binding constant of RR2 for different concentrations of TX-114. (**a**) 0.10 mM; (**b**) 0.13 mM; (**c**) 0.18 mM; (**d**) 0.19 mM.

From the differential absorbance data, the binding constant of RR2 in the MMS is calculated using Equation (1). The different variables of the Benesi–Hildebrand equation are plotted in Figure 11.

**Figure 12:**
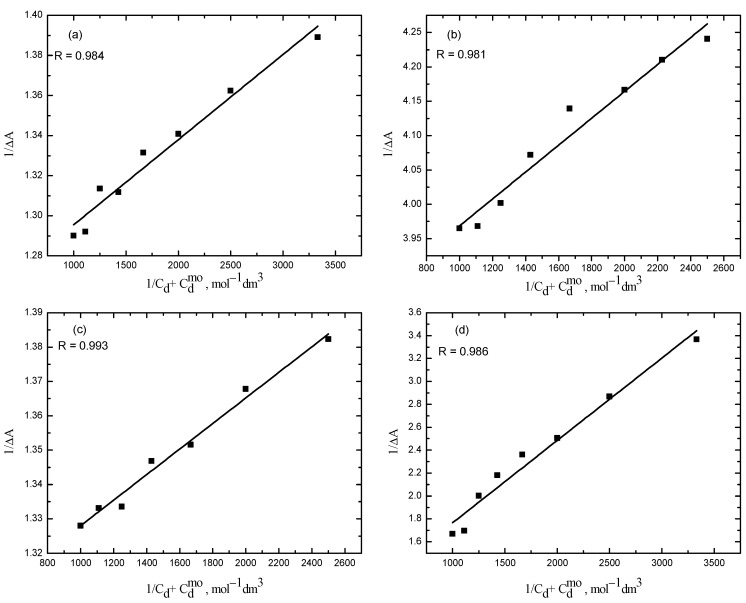
Plots for the calculation of the partition coefficient for different concentrations of TX-114. (**a**) 0.10 mM; (**b**) 0.13 mM; (**c**) 0.18 mM; (**d**) 0.19 mM.

The partition coefficient that describes the partitioning of RR2 in the aqueous and micellar phase is determined using the Kawamura model (Equation (2)) at four different concentrations of TX-114 in the post micellar region of the CPC. The plots between 1/Δ*A* and 1/($C_{d}+C_{s}^{mo}$) are shown in Figure 12.

Table 2 shows the obtained values of the binding constants and partition coefficient for different concentrations of TX-114. The larger values suggest that RR2 binds strongly with the micelles of the CPC–TX-114 MMS. However, for 0.19 mM TX-114, the binding is significantly poor. This could reasonably be attributed to weak electrostatic forces between the RR2 and CPC micelles at the highest concentration of TX-114 in the current work. This happens because, at a high concentration of TX-114, the ionic character of the CPC surfactant decreases [31].

The above data also suggest a significant partitioning of RR2 between the aqueous and micellar phases. Further, a trend similar to the *K_b_* values is obtained for the *K_x_* values. The large values of the Gibbs free energies of binding and partitioning in the presence of a nonionic surfactant indicate that solubilization occurs to a larger extent in the mixed micellar system than in single micelles. However, at the highest concentration (0.19 mM) of TX-114, a decrease in binding and partition constant is observed due to the decrease in the ionic character of the ionic surfactant. So, it can be inferred from the present study that the optimum concentration for the solubilization of RR2 in the CPC–TX-114 MMS is 0.18 mM of TX-114 (Table 2).

### 2.2. Electrical Conductivity Measurements

Electrical conductivity, κ, is a very reliable technique to determine the CMC of a surfactant. Through the CMC values at different temperatures, various thermodynamic parameters can be calculated. These parameters can then be used to determine the stability of the micellar system.

Gibbs free energy of micellization is calculated from the equation given below (Equation (5)) [12,17].

(5) $$\Delta G_{mic}^{{}^{\circ}}=(2-\alpha )RTlnX_{cmc}\;\;\;\;\;$$

**Figure 13:**
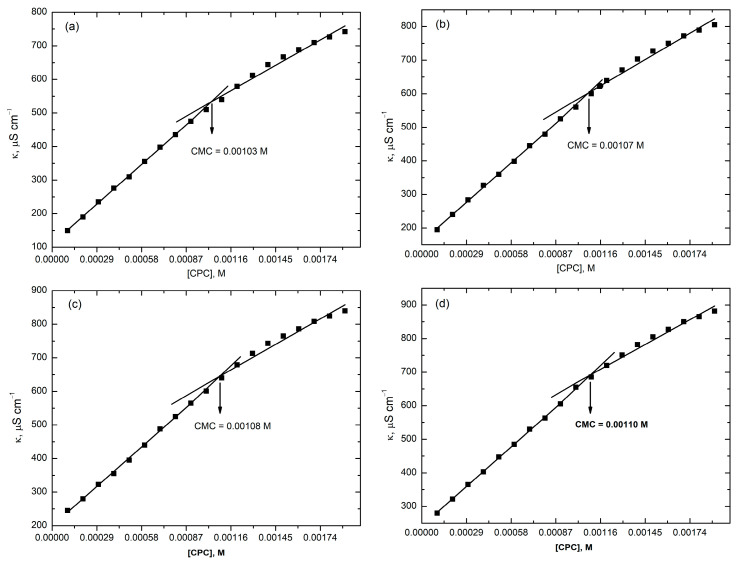
Conductivity versus concentration plot of CPC in the presence of RR2 (1 × 10^−4^ M) at four different temperatures. (**a**) 293.15 K; (**b**) 303.15 K; (**c**) 313.15 K; (**d**) 323.15 K.

**Figure 14:**
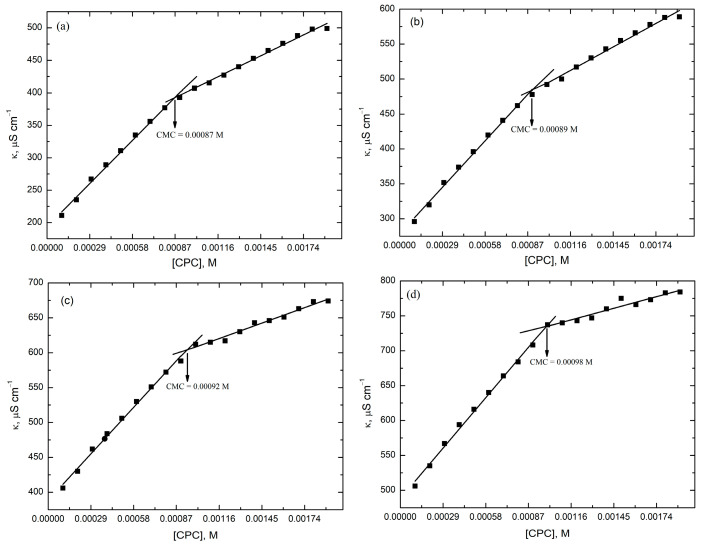
Conductivity versus concentration plot of CPC in the presence of 1 × 10^−4^M RR2 and 0.13 mM TX-114 at four different temperatures. (**a**) 293.15 K; (**b**) 303.15 K; (**c**) 313.15 K; (**d**) 323.15 K.

Here, α is the degree of dissociation, which is the ratio of the slope of post-micellar region to the slope of pre-micellar region of the plots of κ versus concentration of surfactants (Figure 13 and Figure 14), *R* is the general gas constant, *T* is the absolute temperature and $X_{cmc}$ is the critical micelle concentration expressed in mole fraction.

Equations (6) and (7) are used to compute the enthalpy of the micellization $(\Delta H_{mic}$) and the entropy of the micellization (Δ*S_mic_*), respectively [12,17]. The plot between the Log*X_cmc_* and temperature provides the straight line having a slope equal to ∂logXcmc∂T. This value of the slope, substituted in Equation (6), provides the enthalpy of the micellization $(\Delta H_{mic}$) and the subsequently entropy of the micellization (Δ*S_mic_*) can be calculated using Equation (7).

(6) ΔHm=−2.3032−αRT2∂logXcmc∂T

(7) $$\Delta S_{mic}=\frac{\Delta H_{mic}-\Delta G_{mic}}{T}$$

The relationship between the electrical conductivity and the concentration of CPC in the presence of RR2 dye at different temperatures is shown in Figure 13 and Figure S6. Electrical conductivity data were used to find the CMCs of CPC at the studied temperatures. The obtained values are shown in Figure 13 and Table 3. Below the CMC, i.e., in the premicellar region, the molecules of CPC show dissociation and a dynamic equilibrium is attained between the undissociated and dissociated molecules and the conductivity increases rapidly with the increasing concentration of CPC. However, after the CMC, the slope is less due to a reduction in the mobility of the micelles and fewer available free ions.

Dyes can increase or decrease the CMC of a surfactant. If the structure of a dye is rigid then it becomes hard for the dye to incorporate into the micelles. Then, the CMC of the surfactant increases and the process of micellization is less favored. The reason behind this can be that the aggregation of the molecules of the dye near the water micelles interface and the entropy is less. However, if the structure of the dye is less rigid then the molecules of the dye adjust easily into the micelles; then, the CMC value decreases because the repulsion between the head groups of the surfactant is decreased. Here, in the present work, the CMC of the CPC slightly increase from 0.0009 M to 0.0011 M. The reason behind this is the interaction between the RR2 with the CPC molecules and the structure breaking effect of the dye. The complex structure of RR2 makes it difficult to arrange itself into the micelles of the CPC; so, the dye molecules arrange themselves to be able to adjust within the micelles. This orientation disfavors the micellization process and the CMC increases. By increasing the temperature, the CMC value increases because the structure of the water molecules is disrupted around the hydrophobic chain of the surfactant more than the hydrophilic moieties [32]. The thermodynamic parameters of the micellization of CPC in the presence of RR2, calculated at four different temperatures, are given in Table 3.

As evident from Table 3, the free energy of micellization (Δ*G°_m_*) changes steadily from −36.1 kJ/mol to −39.9 kJ/mol by increasing the temperature from 293.15 K to 323.15 K, respectively. This shows that at 323.15 K, micellization is more feasible. However, the obtained values of entropies are nearly constant, which indicates the spontaneity and stability of the system.

Figure 14 and Figure S7 show the electrical conductivities of the RR2–CPC/TX-114 system. This data is used to calculate the CMCs of the mixed micellar systems in the presence of RR2 (Figure 14). For the determination of the CMCs of the MMS, different concentrations of CPC were prepared in an aqueous solution of 0.13 mM nonionic surfactant (TX-114) and 1 × 10^−4^ M RR2. The CMCs of the MMS (Figure 14, Table 4) are significantly lower than those of the RR2–CPC system (Figure 13, Table 3) due to the presence of the nonionic surfactant TX-114. In the presence of TX-114, RR2 solubilizes more effectively into the micelles of CPC.

A quick comparison of the data given in Table 3 and Table 4 reveals that in the presence of TX-114, the micellization is easier and more spontaneous at all of the studied temperatures. Moreover, the micellization is more exothermic in the presence of TX-114. When the change in the free energy of the micellization (Δ*G****^°^****_m_*) of both systems is compared, it is clear that with the increase in temperature, the micellization becomes more spontaneous in both systems; however, it is more spontaneous in the mixed micellar systems at all studied temperatures. The degree of dissociation of the counter ion, α, is also reported in Table 4. It can be seen in Table 4 that the value of α is decreasing by increasing temperature; however, at 303.15 K the value increases, which may be due to the structure breaking effect. The enthalpy of the micellization is positive if the water molecules surround the hydrophobic groups rather than hydrating the hydrophilic groups of the surfactants. However, the Δ*H****^°^****_m_* values are negative when the water molecules hydrate the hydrophilic groups. In the present work, the obtained Δ*H****^°^****_m_* values were negative for single as well as mixed micellar systems. However, the values were higher for the mixed micellar system compared to the single micellar system.

The results in Table 3 and Table 4 show that the entropy change of both the single and MMS is positive and increases with the increase in temperature from 293.15 K to 323.15 K, respectively. Table 4 shows that the entropy of the mixed MMS is increased from 0.009 kJ/K mol to 0.139 kJ/K mol by increasing the temperature from 293.15 K to 323.15 K, respectively. From the data presented in Table 3 and Table 4, it is evident that micellization in both the single and mixed micellar systems is entropically favorable. The entropy of the system is increased due to the movement of the surfactant molecules from the aqueous phase into the micellar phase and the weakening of strong interactions between the water molecules and the surfactant’s molecules. Therefore, this phenomenon leads to increases in the entropy of the system due to the destructuring of the water molecules and the vibrational movements of the hydrophobic tail [32,33,34].

## 3. Material and Methods

### 3.1. Reagents

All of the chemicals used in the present work are laboratory grade reagents. CPC (purity ≥ 98 %) was obtained from Sigma Aldrich Co. (St. Louis, MO, USA). Triton X-114 (TX-114, C_18_H_30_O_3_, Mol. Weight 294.43 g/mol, 2-(2-[4-(1,1,3,3-tetramethylbutyl)phenoxy]ethoxy)ethanol) and RR2 of high purity were obtained from RHAWN Co., Ltd. (Shanghai, China). All the chemicals were used without any additional purification. Distilled water was used throughout the presented study as the aqueous phase for the preparation of all solutions.

### 3.2. Procedure

Firstly, dye solutions of different concentrations were prepared. For the UV-visible spectrophotometry, a solution of 10^−4^ mol/dm^3^ (M) concentration was selected as the optimum concentration for further studies. This solution of dye was used as a solvent to prepare the series of CPC solutions; whereas, for the mixed micellar system, the solutions of RR2 and TX-114 were used as a solvent to prepare the solution of CPC from the pre- to post-micellar region. All the spectra were recorded after 24 h of solution preparation to ensure the dynamic equilibrium between the dye and surfactant.

### 3.3. Conductometric Studies

Electrical conductivity was recorded using the InoLab Cond 7110 (WTW, Cologne, Germany) benchtop conductometer connected to a conductivity cell (KLE 325, WTW Germany) consisting of two graphite electrodes. The cell constant of the conductivity cell was 0.84 cm^−1^. A standard solution of KCl was used to calibrate the conductivity cell. Conductivity data were recorded at temperatures of 293.15 K, 303.15 K, 313.15 K, and 323.15 K. Recorded conductivity data at different temperatures were used to determine the CMC of the ionic surfactant and mixed micellar system in the presence of the RR2 dye at the respective temperatures. The CMC values were used to calculate the thermodynamic parameters of the micellization of single and mixed micellar systems [12,17,19,22].

### 3.4. UV-Visible Spectroscopy

UV-visible spectroscopy is the most simple and easily available technique for the study of dye–surfactant interactions. To study the solubilization of the RR2 dye in the mixed micelles of CPC and TX-114, UV-visible spectra were recorded on a computer interfaced with Analytik Jena (Specord 210 Plus) and a double beam UV-visible spectrophotometer. Quartz cuvettes were used to measure the simple and differential absorbance at 298.15 K with an accuracy of ±0.5 K. For the simple absorbance measurements, distilled water was placed in the reference socket, while the dye solution containing the surfactant was placed in the sample socket. For the differential absorbance, the dye solution was placed in the reference cell.

## 4. Conclusions

The interaction of RR2 dye in cationic micelles of CPC and mixed micelles of TX-114 and CPC has been investigated using electrical conductivity measurements and UV-visible spectroscopy. The degree of solubilization in terms of the partition coefficient (*K_x_*), binding constant (*K_b_*), Gibbs free energy of binding (Δ*G****^°^****_b_*), and Gibbs free energy of partitioning (Δ*G****^°^****_p_*) has been studied. The obtained values of *K_x_* and *K_b_* for the respective systems indicated that mixed micelles are more efficient than single micelles for the solubilization of RR2 in aqueous media. The negative values of Δ*G****^°^****_b_* and Δ*G****^°^****_p_* manifested that the process of solubilization is spontaneous in nature for both single micelles and mixed micelles. The different thermodynamic parameters of the micellization, i.e., Δ*H****^°^****_m_*, Δ*S****^°^****_m_* and Δ*G****^°^****_m_*, have been determined from the obtained values of the CMCs at different temperatures using the electrical conductivity method. The negative values of Δ*H****^°^****_m_* suggest that the process of micellization is exothermic in nature. The positive value of Δ*S****^°^****_m_* indicated an increase in the entropy of the single and mixed micellar systems during the process of micellization. It is concluded that the cationic surfactant, CPC, can be used with the nonionic surfactant, TX-114, for the effective removal of RR2 dye from an aqueous solution.

## Figures and Tables

**Table 1:** Thermodynamic parameters, i.e., the binding constant (*K_b_*), partition constant (*K_c_*), partitioning coefficient (*K_x_*), change in standard free energy of binding (Δ*G_b_*) and change in standard free energy of partitioning (Δ*G_p_*) for the RR2–CPC/TX-114 aqueous system.

Dye–Surfactant System	*K_b_* × 10^−3^ (dm^3^/mol)	Δ*G_b_* (kJ/mol)	*K_c_* × 10^−3^ (dm^3^/mol)	*K_x_* × 10^−4^	Δ*G_p_* (kJ/mol)
RR2–CPC	40 ± 2	−26.3 ± 0.3	26 ± 1	1500 ± 2	−34.6 ± 0.3
RR2–TX-114	23 ± 1	−24.8 ± 0.2	2.4 ± 0.5	14 ± 1	−29.3 ± 0.3

**Table 2:** Binding constant (*K_b_*), partition constant (*K_c_*), partition coefficient (*K_x_*), Gibbs energy of binding (Δ*G_b_*) and Gibbs energy of partition (Δ*G_p_*) for the RR2–CPC/TX-114 mixed micellar system.

Conc. of TX-114 (mM)	*K_b_* × 10^−3^ (dm^3^/mol)	Δ*G_b_* (kJ/mol)	*K_c_* × 10^−3^ (dm^3^/mol)	*K_x_* × 10^−4^	Δ*G_p_* (kJ/mol)
0.10	39 ± 3	−26.2 ± 0.2	29 ± 1	163 ± 1	−35.5 ± 0.2
0.13	29 ± 2	−25.5 ± 0.2	19 ± 1	107 ± 1	−34.4 ± 0.2
0.18	52 ± 2	−26.9 ± 0.2	35 ± 1	192 ± 1	−35.9 ± 0.2
0.19	6 ± 1	−21.7 ± 0.2	2 ± 1	8 ± 1	−28.0 ± 0.2

**Table 3:** CMC of CPC, change in the Gibbs energy of micellization (Δ*G****^°^****_m_*), change in the enthalpy of the micellization (Δ*H****^°^****_m_*), change in the entropy of the micellization (Δ*S^°^_m_*), and the degree of dissociation (*α*) of the RR2–CPC at four different temperatures.

Temperature (K)	CMC (mM)	∆*G^°^_m_* (kJ/mol)	Δ*H^°^_m_* (kJ/mol)	Δ*S^°^_m_* (kJ/Kmol)	α
293.15	1.03	−36.1	−1.9	0.116	0.640
303.15	1.07	−37.0	−2.1	0.115	0.646
313.15	1.08	−38.2	−2.2	0.115	0.647
323.15	1.10	−39.9	−2.4	0.117	0.628

Standard uncertainties in *T*, *CMC*, ∆*G^°^_mic_*, ∆*H^°^_mic_*, ∆*S^°^_mic_* and *α* are ±0.15 K, ±0.0001 M, ±0.2 kJ/mol, ±0.1 kJ/mol, ±1 × 10^−3^ kJ/K. mol, and ±1 × 10^−3^, respectively.

**Table 4:** Different thermodynamic parameters, i.e., change in the Gibbs energy of the micellization (Δ*G****^°^****_m_*), change in the enthalpy of the micellization (Δ*H****^°^****_m_*), change in the entropy of the micellization (Δ*S^°^_m_*) and the degree of dissociation (α) of the counter ions in the mixed micellar system (RR2/CPC/TX-114) at four different temperatures.

Temperature (K)	CMC (mM)	∆*G^°^_m_* (kJ/mol)	Δ*H^°^_m_* (kJ/mol)	Δ*S^°^_m_* (kJ/Kmol)	α
293.15	0.87	−32.4	−5.0	0.009	0.484
303.15	0.89	−41.5	−5.2	0.120	0.505
313.15	0.92	−47.6	−6.2	0.132	0.332
323.15	0.98	−51.9	−7.1	0.139	0.230

Standard uncertainties in *T*, *CMC*, ∆*G^°^_mic_*, ∆*H^°^_mic_*, ∆*S^°^_mic_* and *α* are ±0.15 K, ±0.0001 M, ±0.2 kJ/mol, ±0.1 kJ/mol, ±1 × 10^−3^ kJ/K. mol, and ±1 × 10^−3^, respectively.

## Data Availability

No new data were created or analyzed in this study. Data sharing is not applicable to this article.

## Supplementary Materials

The following supporting information can be downloaded at: https://www.mdpi.com/article/10.3390/molecules28093952/s1, Figure S1. Simple absorbance spectra of RR2 as a function of different concentrations of CPC in aqueous solution. Figure S2. Differential absorbance spectra of RR2 as a function of different concentrations of CPC in aqueous solution. Figure S3. Plot for the calculation of the binding constant, Kb, of the RR2–TX-114 system. Figure S4. Plot for the calculation of the partition coefficient, Kc, for the RR2–TX-114 system. Figure S5. Simple absorbance spectra of RR2 in the presence of 0.13 mM TX-114 in the CPC/TX-114 mixed micelles at different concentrations of CPC. Figure S6. Conductivity vs. concentration plot of CPC in the presence of RR2 (1 × 10^−4^ M) at different temperatures. Figure S7. Conductivity vs. concentration plot of CPC in the presence of 1 × 10^−4^ M RR2 and 0.13 mM TX-114 at different temperatures.
