# Supplementary material for: Solubilization of Reactive Red 2 in the Mixed Micelles of Cetylpyridinium Chloride and TX-114
Source: Molecules. 2023 May 8;28(9):3952. doi: 10.3390/molecules28093952 (PMC10180315; doi:10.3390/molecules28093952)
Supplement: Supplementary file 1 [file molecules-28-03952-s001.zip › molecules-2339216-supplementary.pdf]

molecules

Supplementary Materials

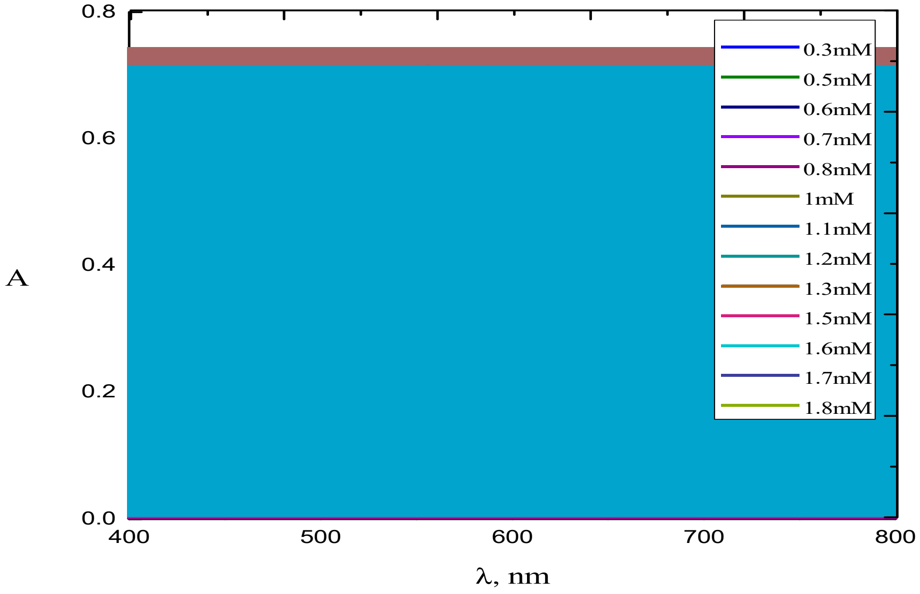

Figure S1. Simple absorbance spectra of RR2 as a function of different concentrations of CPC in aqueous solution.

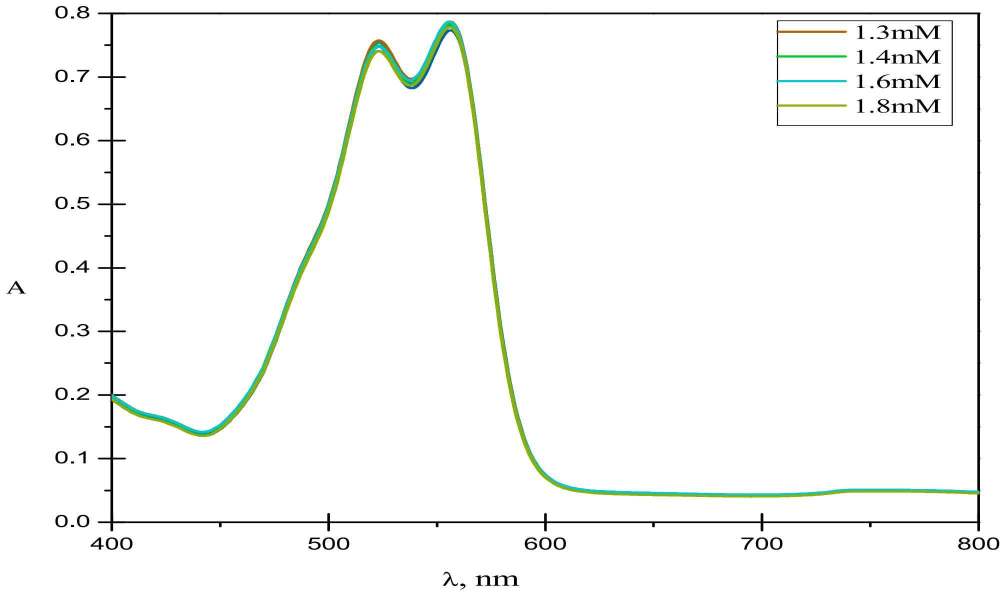

Figure S2. Differential absorbance spectra of RR2 as a function of different concentration of CPC in aqueous solution.

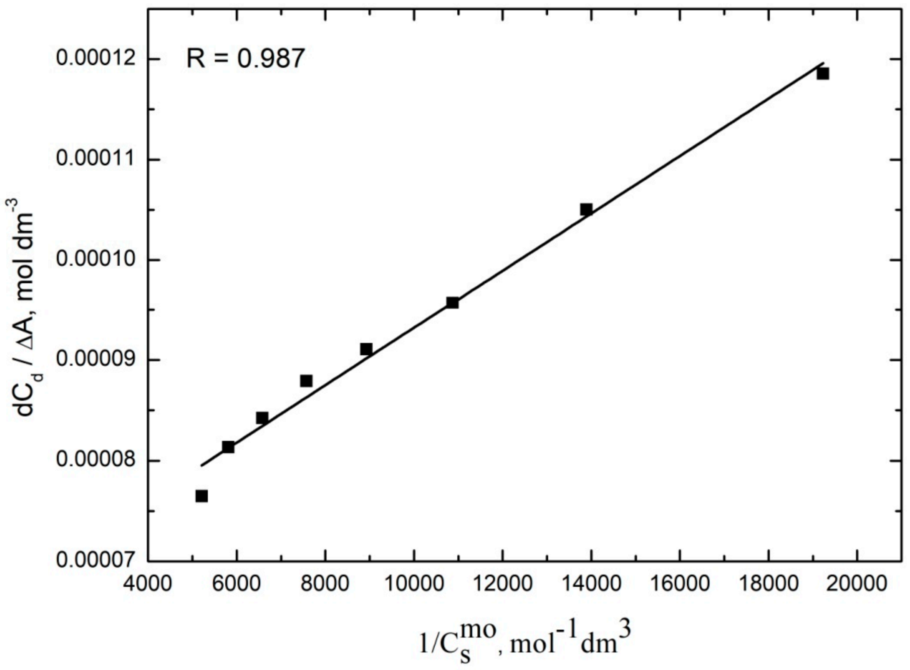

Figure S3. Plot for calculation of binding constant*, K_b*, of RR2-TX-114 system.

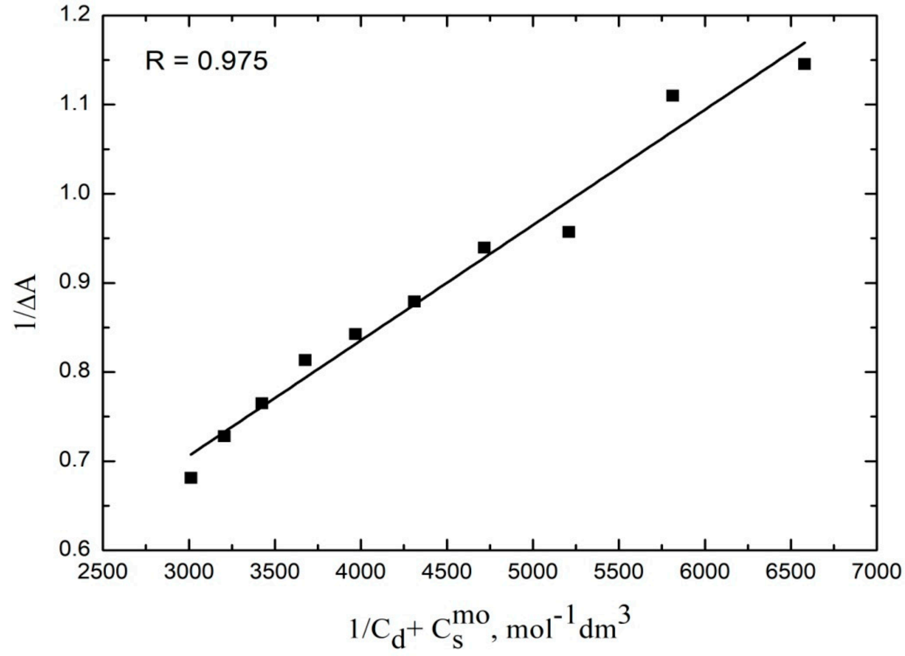

Figure S4. Plot for calculation of partition coefficient, *K_c*, for the RR2-TX-114 system.

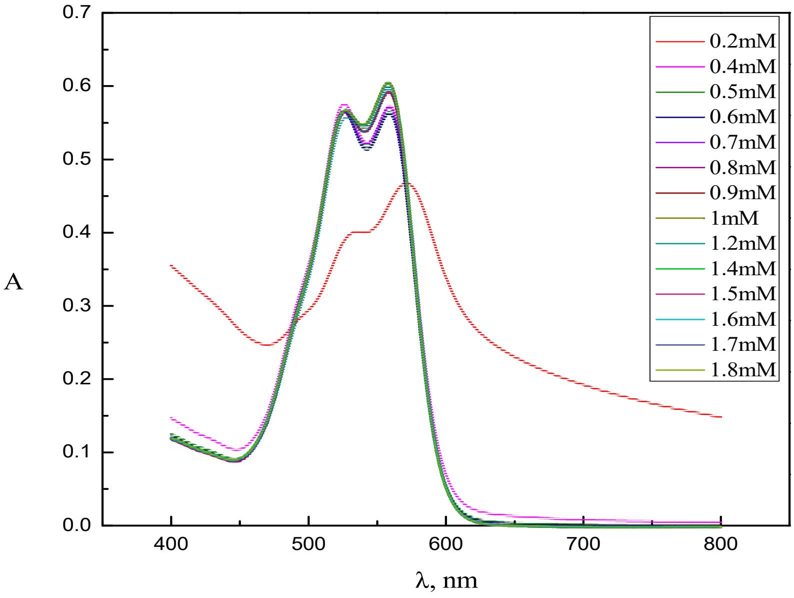

Figure S5. Simple absorbance spectra of RR2 in the presence of 0.13 mM TX-114 in the CPC/TX-114 mixed micelles at different concentrations of CPC.

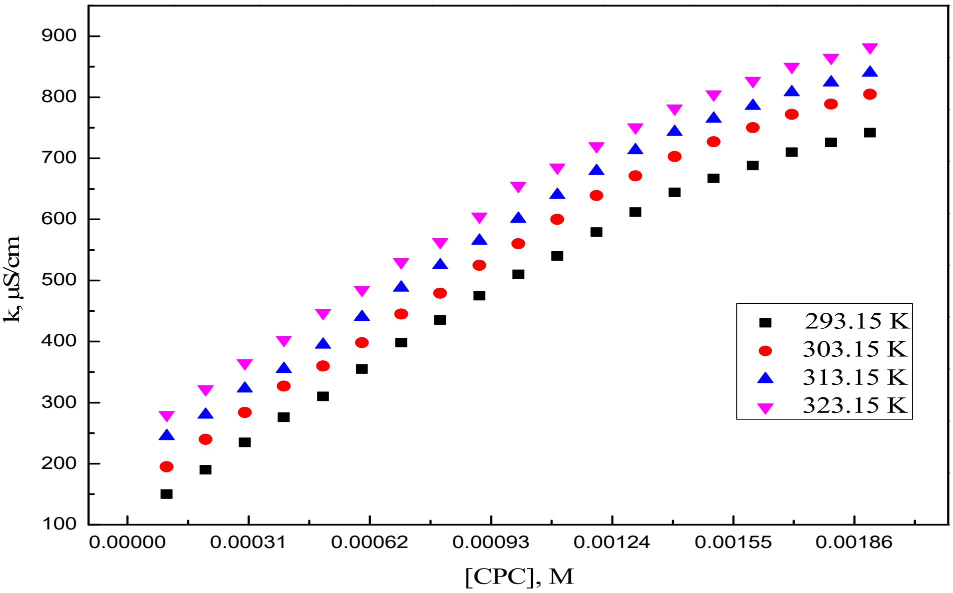

.

FigureS6. Conductivity vs concentration plot of CPC in presence of RR2 (1×10^{-4}M) at different temperatures.

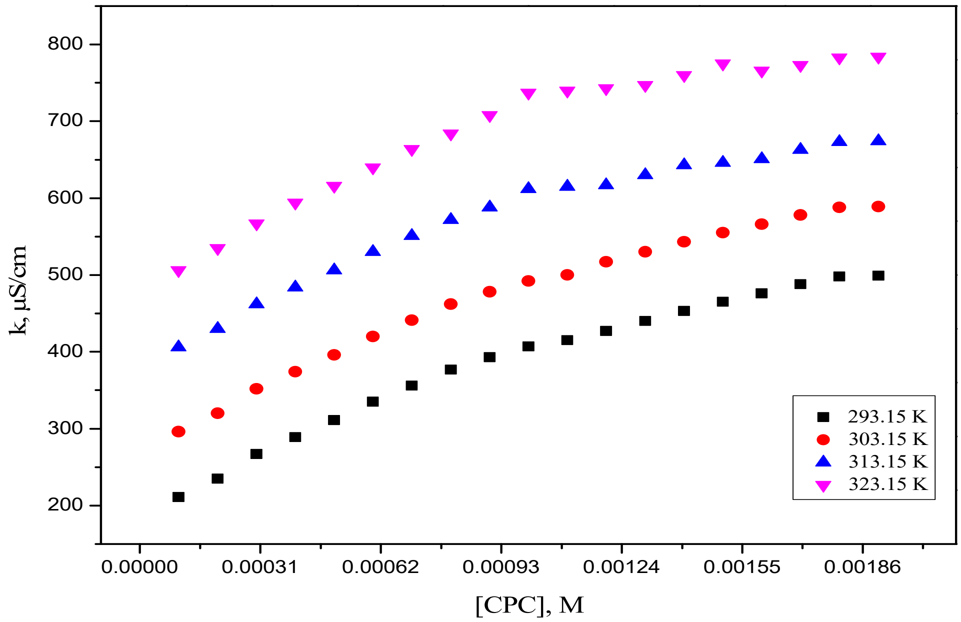

Figure S7. Conductivity vs concentration plot of CPC in presence of 1×10^{-4}M RR2 and 0.13 mM TX-114 at different temperatures.
